# Cysteine residues are essential for dimerization of Hippo pathway components YAP2L and TAZ

**DOI:** 10.1038/s41598-018-21828-6

**Published:** 2018-02-22

**Authors:** Prem Khanal, Zongchao Jia, Xiaolong Yang

**Affiliations:** 1 Department of Pathology and Molecular Medicine, Queen’s University, Kingston, Canada; 2 Department of Biomedical and Molecular Sciences, Queen’s University, Kingston, Canada

## Abstract

Hippo signalling pathway is an emerging signalling pathway that plays important roles in organ size control, tumorigenesis, metastasis, stress response, apoptosis, stem cell differentiation and renewal during development and tissue homeostasis. Recent studies reported that human serine/threonine protein kinase, Mst1, a core component of the Hippo pathway can be activated through formation of homodimer. However, it is still unclear whether or not other components of the Hippo pathway are also regulated through dimerization. Here we provide the first evidence that Hippo components and oncoprotein YAP2L and TAZ can form homodimer *in vitro* and *in vivo* by forming disulphide bond through cysteine residue(s). We have also shown that the homodimers of YAP2L/TAZ are more stable and showed more oncogenic behaviour than their corresponding monomers as revealed by colony formation and cell transformation assay. Since cysteine post-translational regulation plays important roles in redox signalling, tumorigenesis and drug resistance, further studies on the functional effect of this dimerization through post-translational modulation of cysteine residues in YAP2L/TAZ will provide a significant contribution to our understanding of the roles of YAP2L/TAZ in cancer development and therapy.

## Introduction

One of the common properties of the proteins is that they can form dimer or high-order oligomers through self-association^1^. The formation of either intermolecular or intramolecular dimers or oligomers is very important process in protein folding^2^. Proteins usually form dimers or oligomers through specific motifs such as leucine Zipper^3,4^, helix-loop-helix^5^, Ankyrin^6^ and PAS-domain^7^ or disulphide bond formation between cysteines (C)^8^. The dimeric or oligomeric forms of proteins have several impacts on their roles in cellular process^1^. For example, they can increase the activity and stability of proteins^9,10^, help to transport of molecules across cell membranes^11,12^, increase protein binding affinity to DNA and transcriptional activity^13,14^, cause increased cell proliferation, transformation and drug resistance^13,15^, and inhibit cell transformation^16^, Therefore, identification and characterization of novel dimeric/multimeric proteins are very important for fully understanding their functions in different cellular process.

The Hippo signaling pathway, which was initially identified in *Drosophila*, is highly conserved in mammals^17^. This novel signalling pathway is involved in regulation of tissue growth, organ size, cell proliferation, apoptosis, tumorigenesis, mechanotransduction, drug resistance, stem cell renewal and differentiation^18–28^. Serine/threonine (S/T) kinases MST1/2 and LAT1/2, adaptor/scaffold proteins MOB1 and WW45, and two WW domain containing transcriptional co-activators YAP and its paralog TAZ are the main components of the Hippo pathway^24,29–35^. YAP and TAZ are the main downstream effector of Hippo pathway and are involved in cell proliferation, drug resistance and many other tumorigenic processes by transactivation of downstream genes (CTGF, Cy61, BMP4, etc.) in the nucleus through transcription factor TEAD or Smad^23,35–39^. Activation of LATS1/2 by upstream members (MST1/2, MOB1, and WW45) of the Hippo signalling cascade phosphorylates and inactivates YAP and TAZ by promoting either their cytoplasmic retention or degradation, preventing them from transactivating downstream genes in the nucleus^40,41^.

Many studies have already shown the different mechanisms which leads to either increased or decreased levels or activity of YAP/TAZ and their importance in cancer biology. For example, while SCF^β-TRCP^ E3 ubiquitin ligase decreases the stability of YAP and TAZ by ubiquitin-mediated degradation^27,42^, PP1A and ASPP2 increases the TAZ activity by antagonizing its negative regulator LATS kinase through TAZ dephosphorization^43^. In addition, it has been reported that one of the major components of the Hippo pathway, MST1 undergoes dimerization, which increases its kinase activity^44,45^. However, whether and how the levels or activities of other components of the Hippo pathway are also regulated through dimerization are largely unknown. In this study, we have shown that both YAP and TAZ undergo dimerization through cysteine residues, resulting in increase in their stability which leads to enhanced tumorigenesity of H460 lung cancer cells. This finding provides novel mechanism by which YAP and TAZ are regulated and have significant implication in our understanding the roles of YAP and TAZ in cancer.

## Results

### YAP2L isoform forms dimer *in vitro* and *in vivo*

**Figure 1:**
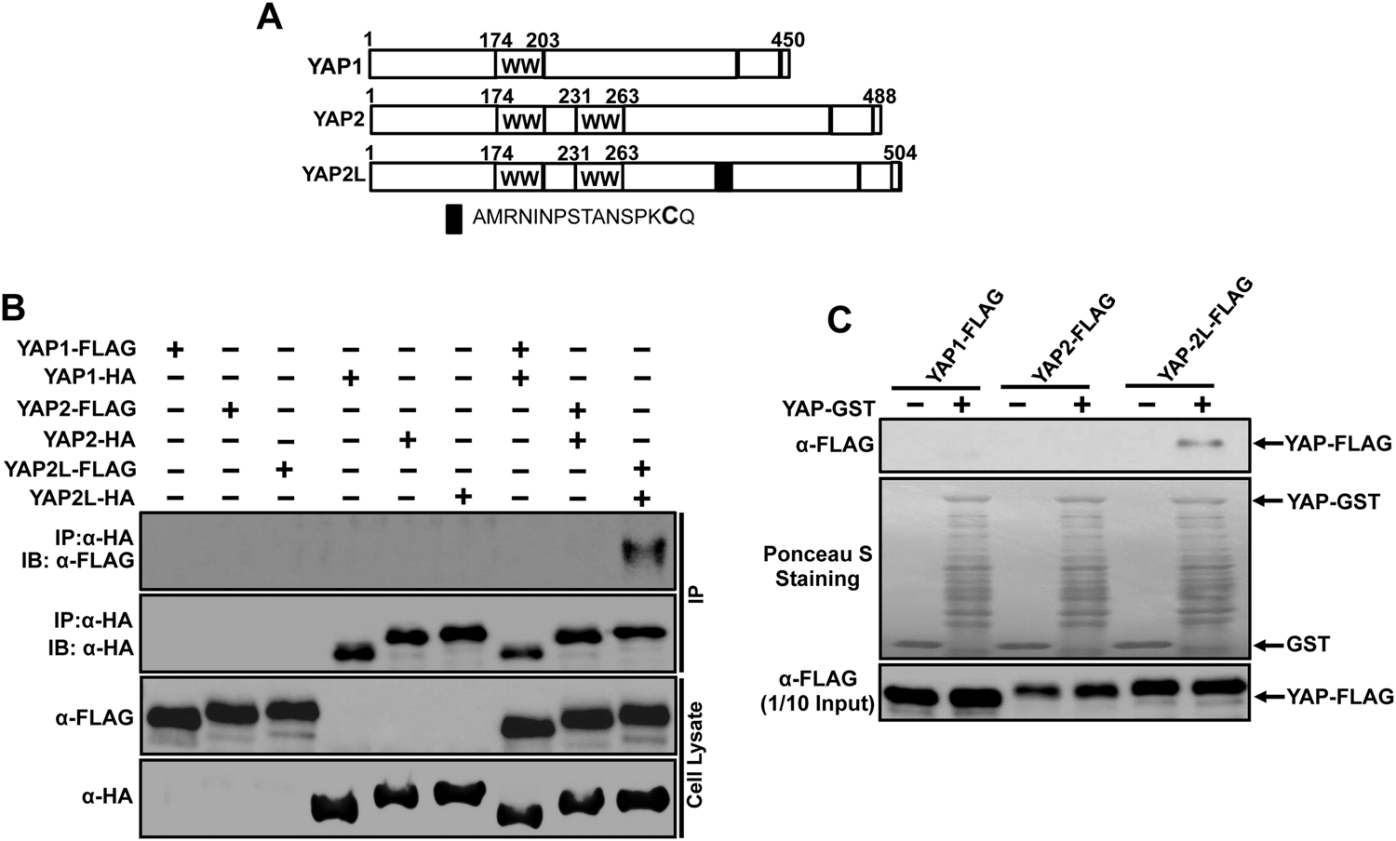
*In vitro* and *in vivo* dimerization of YAP2L isoform. (**A)** Schematic representation of YAP1, YAP2 and YAP2L isoforms. (**B)** YAP2L isoform form dimer *in vivo*, HEK293 cells were transfected with HA or FLAG-tagged-YAP1, -YAP2 and -YAP2L plasmids alone or together. The cells were harvested in 1%NP-40 lysis buffer. After checking the expression level of HA or FALG- tagged-YAP1,-YAP2 and-YAP2L, equal amount of cell lysates were subjected to co-immunoprecipitation assays using anti-HA antibody and immublotting analysis were performed using anti-FLAG or anti-HA antibody respectively. (**C)** YAP2L isoform form dimer *in vitro*, 200 µg of cell lysates from FLAG-tagged-YAP1,-YAP2 and -YAP2L plasmids transfected HEK293 cells were precleared with 50% GSB beads overnight at 4^◦^C. After then, supernatants were mixed with 5 µg of GST or YAP-GST and incubated for 2 hrs followed by addition of 20 μl of 50% GSB beads for another 1 hr. The beads were then washed, eluted by 2XSDS sample buffer and subjected to western blotting against anti-FLAG antibody. Ponceau-S staining shows the equal amount of fusion protein used for pull down. 1/10 input (20 µg) represents 1/10 of protein lysate used for pull down.

The YAP (Yes associated protein 1) oncogene, a major component of hippo signalling pathway, is a transcriptional coactivator of target genes and involved in cell proliferation and survival^28^. Since YAP protein has eight isoforms in human^46^ and there is no antibody to distinguish each isoform, we chose three most commonly studied isoforms of YAP (YAP1, YAP2 and YAP2L) (Fig. 1A) and tested them individually by expressing each isoform in cells. We first carried out a co-immunoprecipitation assay among the three different isoforms of YAP. HA or FLAG-tagged YAP1, YAP2 and YAP2L plasmids were transfected alone or together into HEK293 cells. The total cell lysates were immunoprecipitated with anti-HA antibody and blotted with anti-FLAG antibody. Interestingly, we found that YAP2L, a longest isoform among the three isoforms was found in the immune complex indicating that only YAP2L isoforms forms dimer *in vivo* (Fig. 1B). Next, we further confirmed this interactions using GST-pull down assays. Cell lysates from FLAG-tagged -YAP1, -YAP2 and -YAP2L expression vectors transfected HEK293 cells were used for GST-pull down assays. As expected, only YAP2L was able to bind with YAP-GST (Fig. 1C). In summary, these experiments show that only YAP2L isoforms forms homodimer *in vitro* and *in vivo*.

### Cysteine residue is essential for dimerization of YAP2L

**Figure 2:**
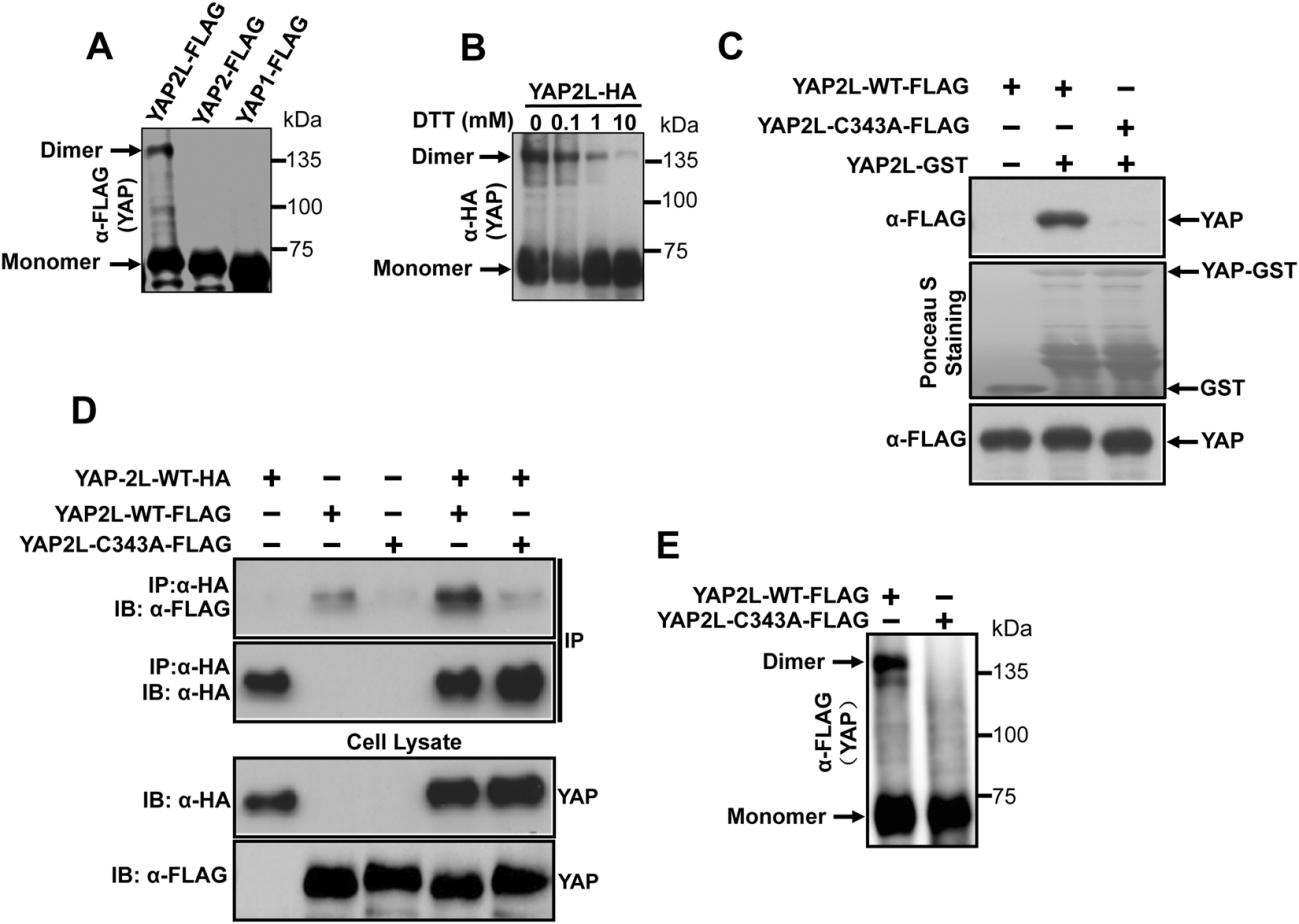
YAP2L undergoes dimerization through cysteine residue. (**A**) YAP2L isoform forms dimer, 15 µg of protein lysates from FALG-tagged-YAP1,-YAP2 and-YAP2L overexpressed HEK293 cells were separated by non- reducing SDS-PAGE and blotted against anti-FLAG antibody. (**B)** DTT treatment disturb the dimerization of YAP2L, 15 µg of protein lysates from HA-tagged-YAP1,-YAP2 and-YAP2L overexpressed HEK293 cells were treated with indicated amount of DTT (Dithiothreitol) and separated by non- reducing SDS-PAGE and blotted against anti-HA antibody. (**C)** Cysteine is necessary for dimerization of YAP2L *in vitro*, 200 µg of protein lysates from FLAG-tagged-YAP2L-WT or -YAP2L-C343A plasmids transfected HEK293 cells were subjected to GST-pull down assays as mentioned above. 5 µg of GST or YAP-GST were used for pull down, immunoblotting was carried using anti-FLAG antibody. (**D)** Cysteine is important for dimerization of YAP2L *in vivo*, co-immunoprecipitation assay was carried was as mentioned above, in brief protein lysate from FLAG-tagged-YAP2L-WT or -C343A and HA-tagged-YAP2L-WT alone or together transfected HEK 293 cells were subjected to immunoprecipitation using anti-HA antibody and the interaction was confirmed by immunoblotting against anti-FLAG and HA antibody respectively. (**E)** YAP2L-C343A mutant doesn’t form dimer, 15 µg of cell lysates from FLAG-tagged-YAP2L-WT or -C343A overexpressed HEK293 cells were separated by non- reducing SDS-PAGE and blotted against anti-FLAG antibody.

**Figure 3:**
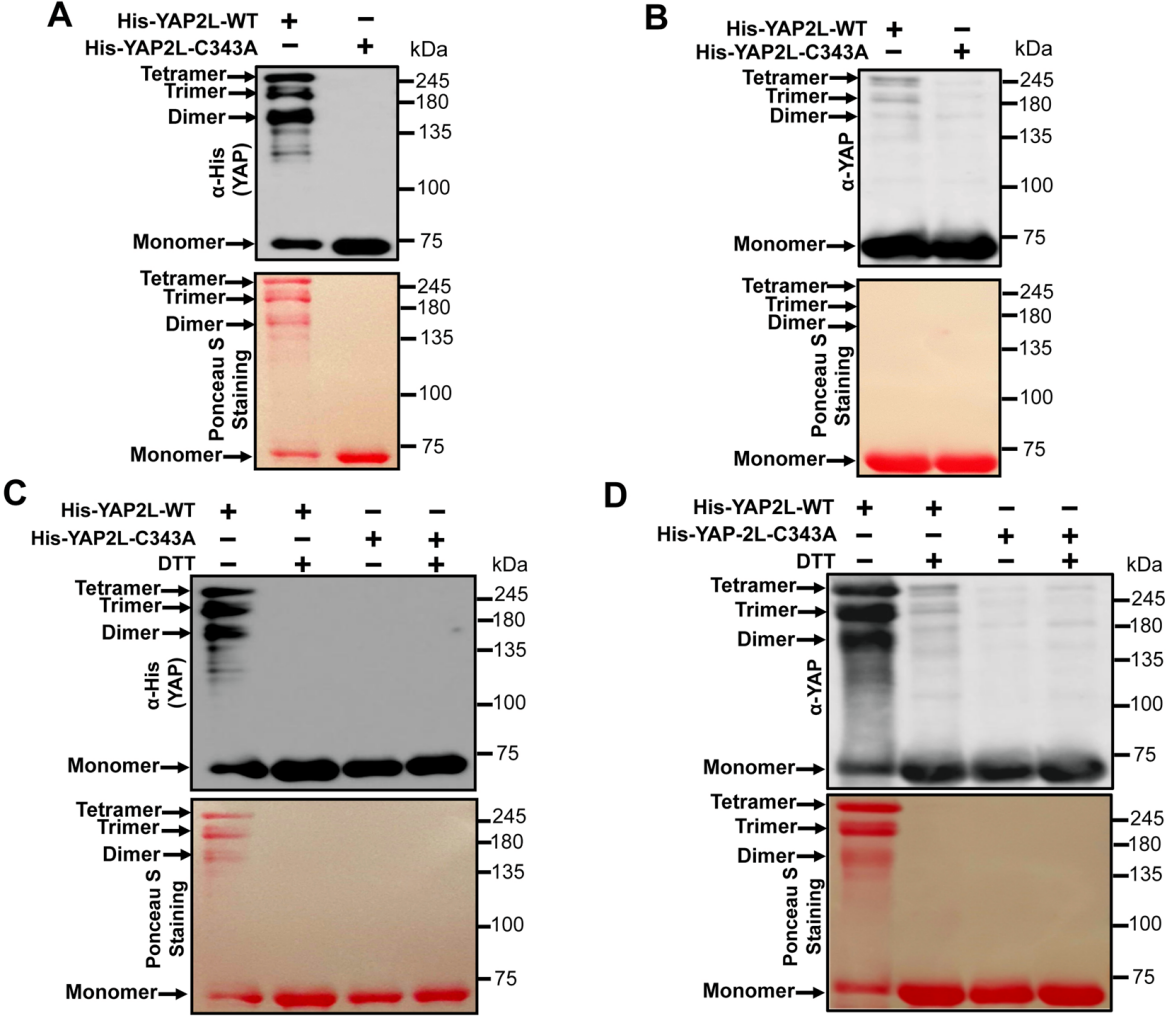
Importance of cysteine residue in dimerization of purified YAP2L. (**A**) YAP2L-C343A mutation loss the ability to form dimer of purified YAP2L, 1 µg of purified His-YAP2L-WT or -C343A mutants were run in non-reducing SDS-PAGE and blotted against anti-His-HRP antibody. (**B**) YAP2L-WT couldn’t form dimer under reducing condition, 1 µg of purified His-YAP2L-WT or -C343A mutants were run in reducing SDS-PAGE and blotted against anti-YAP antibody. (**C** and **D)** DTT treatment inhibit the dimerization of YAP2L-WT, 1 µg of purified His-YAP2L-WT or -C343A mutants were treated with 10 mM of DTT and run in non-reducing SDS-PAGE and blotted against anti-His-HRP (**C**) or anti YAP (**D**) antibody respectively, ponceau-S-staining shows the amount of protein in membrane.

To further explore this specific interaction of YAP2L isoforms, we analyze the sequence of these three isoforms and found that there is one cysteine residue in YAP2L which is not present in YAP1 and YAP2 (Fig. 1A). Next, to explore the possibility of involvement of this cysteine residue in dimerization of YAP2L, we transfected HEK293 with FLAG-tagged-YAP1,-YAP2 and -YAP2L plasmids and cell lysates were run in non-reducing (no DTT) SDS-PAGE. Interestingly, the result showed that YAP2L (70 kDa) has an extra band at exactly at the size of dimer (140 kDa) indicating the possibility of involvement of cysteine in dimerization of YAP2L (Fig. 2A). Since, cysteines are well known for forming the inter- or intra-molecular dimers through the formation of disulphide bond and reducing reagent DTT can disrupt disulfide bonds in protein, we next checked whether the dimerization of YAP2L is disrupted by DTT. Surprisingly, the treatment of DTT inhibited the dimerization of HA-YAP2L in a dose dependent manner (Fig. 2B), indicating that YAP2L forms dimer by forming the disulphide bond between the cysteine residues. Next, in order to check whether this cysteine residue of YAP2L is indeed necessary for this dimerization, we mutate the cysteine of YAP2L to alanine (YAP2L-C343A) and carried out GST-pull down assay using the lysate from HEK293 cells transfected with FLAG-tagged-YAP2L-WT or - C343A mutant and YAP2L-GST fusion protein purified from BL-21 bacteria. As expected, only YAP2L-WT was found to interact with YAP2L-GST and mutation of C343A completely abolished this interaction (Fig. 2C). This was further verified with Co-IP assay using lysates that were transfected with FLAG-tagged YAP2L-WT or -C343A and either HA-tagged YAP2L-WT alone or together (Fig. 2D). Involvement of cysteine residue in the formation of YAP2L dimer was further confirmed by running the FLAG-tagged-YAP2L-WT or -C343A overexpressed HEK293 cell lysates in non-reducing (no DTT) SDS-PAGE, which showed an existence of a dimer band in case of YAP2L-WT only (Fig. 2E). This result was further confirmed by using purified His-tagged-YAP2L-WT or-YAP2L-C343A mutant. For this, 1 μg of purified His-tagged-YAP2L-WT or-YAP2L-C343A mutant were run in non-reducing (no DTT) SDS-PAGE and blotted against anti-His antibody. As expected, only the YAP2L-WT showed a band around the 140 kDa, exactly the same size of dimer of YAP (Fig. 3A). Moreover, other bands were also seen above this dimeric band suggesting the possibility of oligomerization (trimer: 210 kDa; tetramer: 280 kDa) of YAP2L *in vitro*. Next, we checked whether this oligomerization of YAP2L-WT is disrupted under reducing condition. The results showed that purified His-YAP2L-WT reduced its ability to oligomerize when run in reducing SDS-PAGE (Fig. 3B). In order to further confirm the involvement of cysteine residue in the dimerization of YAP2L, 1 μg of purified His-tagged-YAP2L-WT or-YAP2L-C343A mutant were run in non-reducing SDS-PAGE in the presence/absence of 10 mM DTT and blotted against anti-His or anti-YAP antibody respectively (Fig. 3C and D). As before, we found that the dimer formed by purified YAP2L-WT was also inhibited in the presence of DTT indicating that this dimerization of YAP2L is due to the presence of disulphide bond between cysteine residues. Taken together, these data strongly suggest that cysteine residue is responsible for dimerization of YAP2L.

### Cysteine residues are essential for dimerization of TAZ

**Figure 4:**
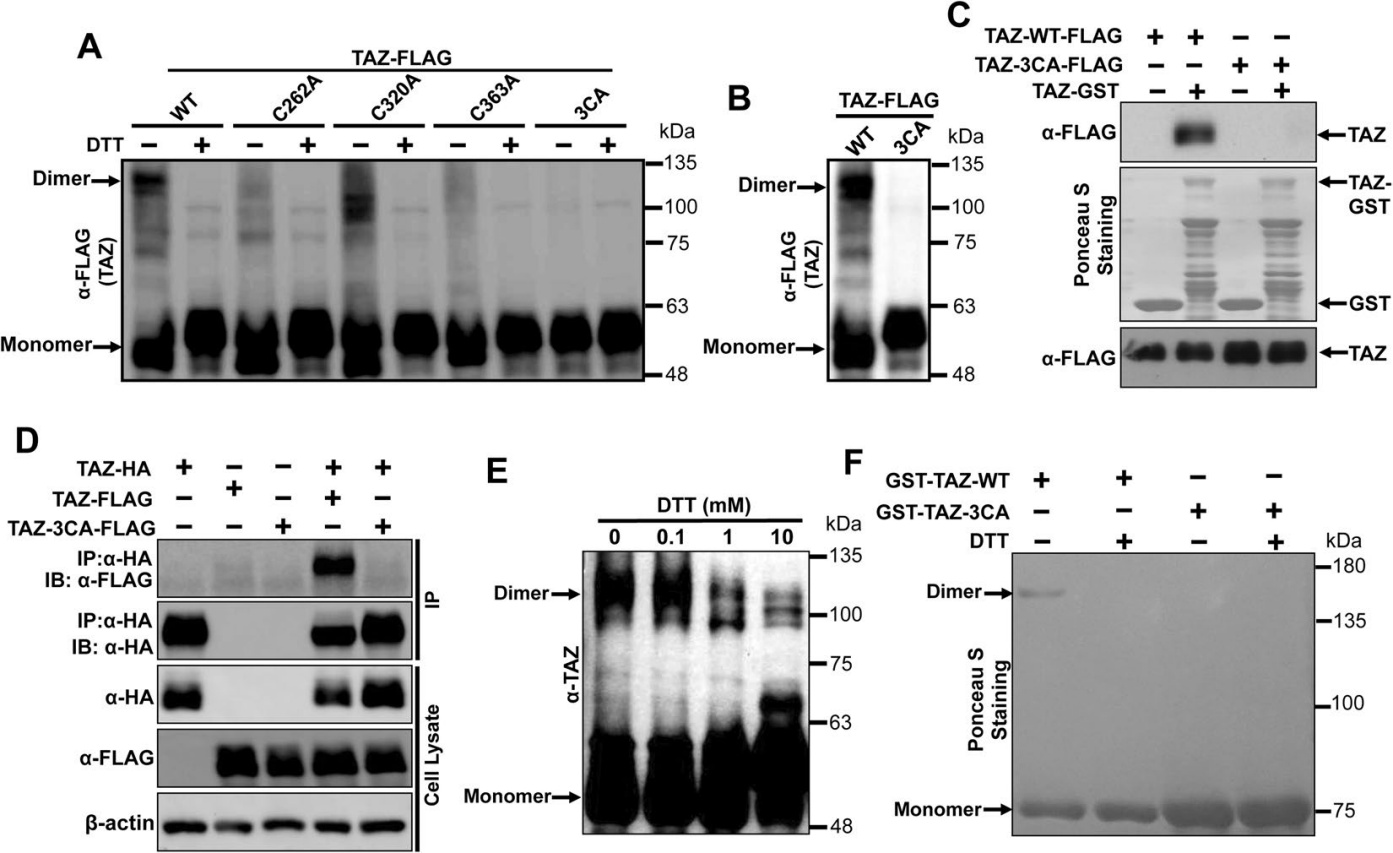
Dimerization of TAZ needs cysteine residues. (**A)** DTT treatment inhibit the dimerization of TAZ, 15 µg of protein lysates from FLAG-tagged-TAZ-WT,-C262A,-C320A,-C363A and-3CA overexpressed HEK293 cells were treated with 10 mM of DTT (Dithiothreitol) and separated by non-reducing SDS-PAGE and blotted against anti-FLAG antibody. (**B)** Mutation of all 3 cysteines of TAZ to alanine (TAZ-3CA) inhibit the dimerization of TAZ, protein lysates from FLAG-tagged-TAZ-WT or -TAZ-3CA overexpressed HEK293 cells were separated by non- reducing SDS-PAGE and blotted against anti-FLAG antibody. (**C)** TAZ-3CA mutant abolish the *in vitro* dimerization of TAZ, GST-Pull down assay was carried out as mentioned above using FLAG-tagged-TAZ-WT and -TAZ-3CA overexpressed cell lysates from HEK293 cells. 5 µg of GST or TAZ-GST were used for pull down, immunoblotting was carried using anti-FLAG antibody. (**D)** Cysteines residues are important for *in vivo* dimerization of TAZ, CO-IP assay was carried out using the 200 µg of FLAG-tagged-TAZ-WT or-TAZ-3CA and HA-TAZ-WT alone or together overexpressed cell lysate from HEK293 cells. Anti-HA antibody was used for immunoprecipitation and immunoblotting was carried out using anti-FLAG and anti-HA antibodies respectively. (**E**) Dimerization of endogenous TAZ, 30 µg of cell lysates from H460 NSCLC cell lines were treated with increasing concentration of DTT as indicated and immunoblotting was carried out using non reducing SDS-PAGE and anti-TAZ antibody. (**F**) Treatment of DTT disturbed the dimerization of purified TAZ, 1 µg of purified GST-TAZ-WT or -TAZ-3CA were treated/not treated with 10 mM of DTT and were run in non-reducing SDS-PAGE and stained by Ponceau-S.

Since TAZ, a paralog of YAP^47^, show similar characteristics towards cell proliferation, cell transformation and both of them are key downstream effectors of the Hippo pathway^48^, we were interested to check whether the cysteine residue is also important for dimerization of TAZ. For this, we first mutated all three cysteines of human-TAZ individually or together into alanine (A) and HEK293 cells were transfected with FLAG-tagged-TAZ-WT, -TAZ-C262A, -TAZ-C320A, -TAZ-C363A or -TAZ-3CA (C262A, C320A andC363A) expression vectors. The resulting cell lysates were separated by non-reducing SDS-PAGE in the presence/absence of 10 mM DTT. Consistently, the result showed that TAZ also form homodimer through cysteine residues which is disrupted in the presence of DTT (Fig. 4A). Although single cysteine has various effects on dimerization, mutation of all three cysteine residues completely abolished TAZ dimerization (Fig. 4A). We further confirmed the importance of all these three cysteines residues of TAZ in its dimerization by running the FLAG-tagged-TAZ-WT of -TAZ-3CA overexpressed HEK2393 cell lysates in non-reducing SDS-PAGE (Fig. 4B). Next, we performed GST-pull down assay to check the effect of mutation of all three cysteine residues of TAZ in its dimerization. When GST pull-down assay was carried out with cell lysates expressing FLAG-tagged TAZ-WT or TAZ-3CA and TAZ-WT-GST fusion protein purified from bacteria, only the TAZ-WT was found to bind with TAZ-GST (Fig. 4C). This was further verified with Co-IP assay using lysates that were transfected with FLAG-tagged TAZ-WT or TAZ-3CA alone or together with HA-tagged TAZ-WT (Fig. 4D). Next, since TAZ expresses only a single isoform in cells, we were able to test whether endogenous TAZ also form dimer in cells. When the whole cell lysates from H460 cells were run on non-reducing SDS-PAGE in the presence of increasing doses of DTT, the dimerization of endogenous TAZ (monomer: 50 kDa; dimer: 100 kDa) was found decreased with increasing doses of DTT (Fig. 4E). We also further confirmed the dimerization of TAZ using purified GST-TAZ-WT or TAZ-3CA-GST. When 1 μg of purified TAZ-WT-GST or TAZ-3CA-GST were resolved by non-reducing SDS-PAGE, only GST-TAZ-WT showed a band exactly at the dimer size (TAZ-GST monomer: 77 kDa; dimer: 154 kDa) of TAZ, indicating that TAZ indeed form homodimer (Fig. 4F). To further confirmed the importance of cysteine residues of TAZ in its dimerization, 1 μg of purified GST-TAZ-WT or -TAZ-3CA were either treated or not treated with10 mM DTT and run on non-reducing SDS-PAGE. As expected, we found that DTT treatment abolished formation of TAZ-WT-GST dimer *in vitro* (Fig. 4F). In summary, all these results confirm that TAZ also formed homodimer through cysteine residues.

### Disruption of YAP and TAZ dimerization reduces their stability

**Figure 5:**
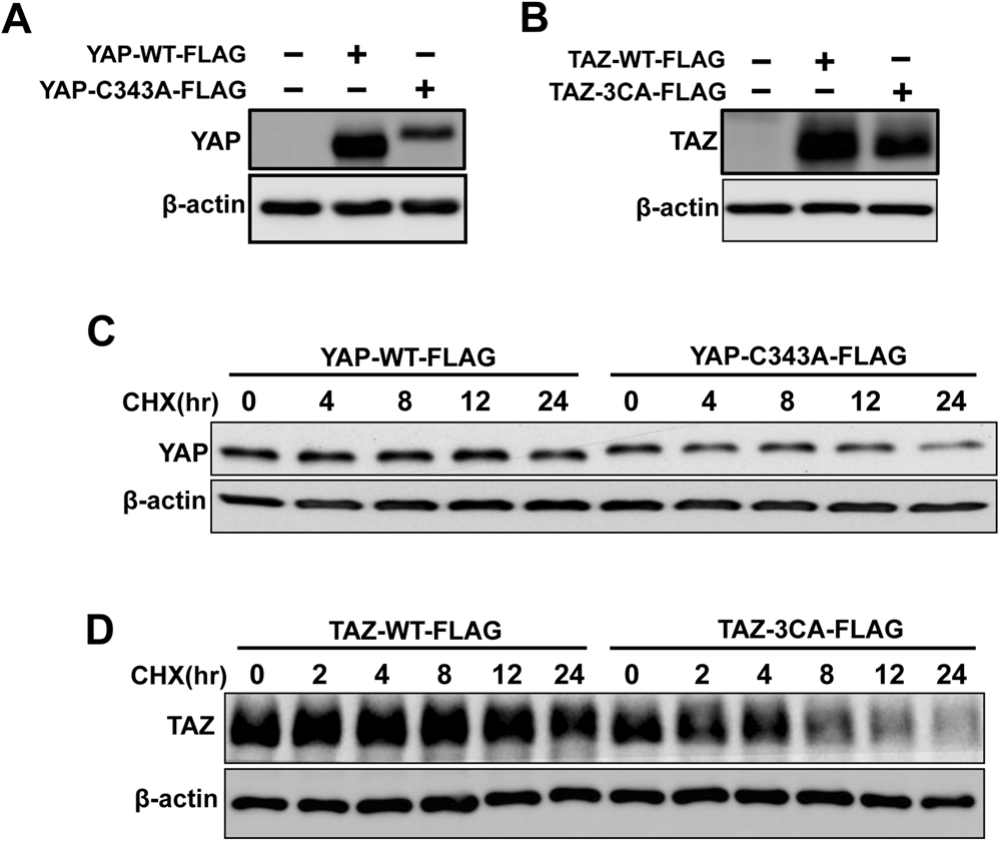
Dimerization of YAP/TAZ increases their stability. (**A** and **B**) low expression of YAP/TAZ cysteines mutants, HEK293 cells were transfected with equal amount of FLAG-tagged-YAP2L-WT or,-YAP2L-C343A (**A**), -TAZ-WT or-TAZ3CA (**B**) separately and western blotting was performed to check the expression of different proteins. (**C** and **D**) less stability of YAP/TAZ cysteine mutants, HEK293 cells were transfected with equal amount of FLAG-tagged-YAP2L-WT or,-YAP2L-C343A (**C**), -TAZ-WT or-TAZ3CA (**D**) separately and after 24 hrs, cells were treated with 100 µg/ml cycloheximide and proteins were extracted at different time intervals as indicated in figure. Western blotting was carried out to check the expression of proteins.

Since it is reported that the dimerization/oligomerizations of proteins increases their stability^1^, we examined whether the dimerization of YAP or TAZ increases their stability. As expected, when equal amount of FLAG-tagged YAP2L-WT/-C343A or TAZ-WT/-3CA plasmids were transfected into HEK293 cells, we found that the expression level of YAP2L-WT or TAZ-WT was always higher as compare to their corresponding cysteine mutants YAP2L-C343A or TAZ-3CA, respectively (Fig. 5A and B). To examine whether the reduced levels of cysteines mutants is due to their decreased turnover half-lives, we performed cycloheximide chase experiments. For these assays, FLAG-tagged-YAP2L-WT/-C343A or -TAZ-WT/-3CA plasmids were transfected in to HEK293 cells and after 24 hr of transfection protein synthesis was inhibited by the addition of cycloheximide. The proteins were extracted at different time intervals as indicated and the expression of protein was examined by western blotting using reducing SDS-PAGE. Consistently, the results showed that cysteine mutants are degraded more rapidly by cycloheximide treatment as compared to their corresponding wild type (Fig. 5C and D). Altogether, these results confirm that dimerization of YAP or TAZ increase their protein stability.

### Dimerization of YAP/TAZ increases their oncogenic function

**Figure 6:**
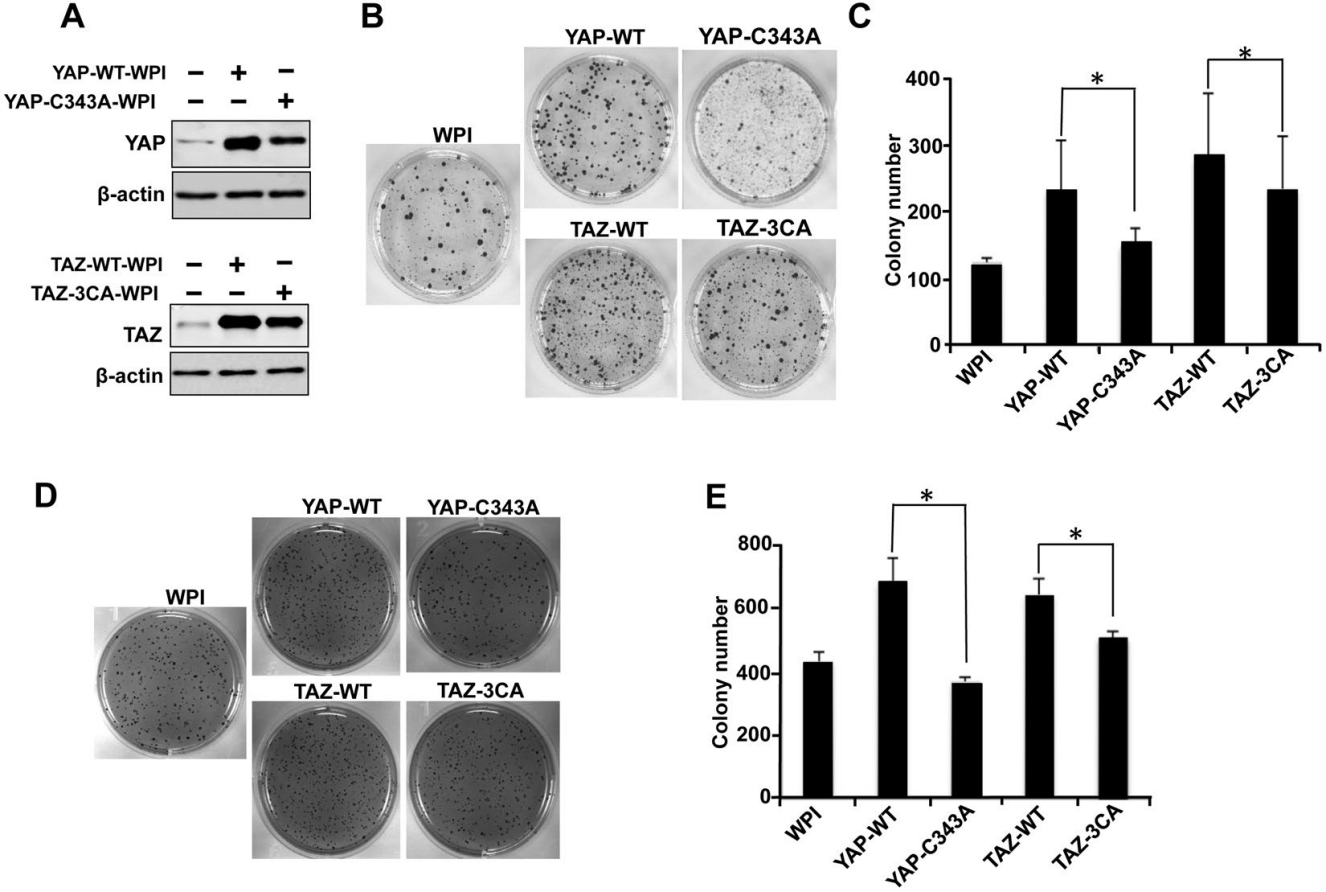
Dimerized YAP/TAZ are more oncogenic than their corresponding monomers. **(A)** Expression level of YAP2L-WT/C343A and TAZ-WT/TAZ-3CA in H460 stable cell lines. (**B** and **C**) Colony formation assay using different stable H460 cell lines. In brief 500 cells/plate of H460-WPI,-HA-YAP2L-WT,-HA-YAP2L-C343A,-HA-TAZ-WT or -HA-TAZ-3CA stable H460 cell lines were plated separately in 60 mm plates in triplicate. The media was refreshed at every 3–4 days. After 10 days, staining and analysis of colony was done as described as in experimental procedures and colony numbers were counted by Bio-Rad Gel Doc System (Bio-Rad, Mississauga, Canada). Data are shown as means ± S.D. “*” Represent significant difference (*P* < 0.05) in *t*-test. (**D** and **E)** Soft Agar Assay was performed as described as in experimental procedures. In brief, 2 × 10^3^ H460-WPI, -YAP-WT,-YAP-C343A, -TAZ-WT and -TAZ-3CA -H460 stable cells were plated for soft agar in 6-well plates (triplicate). Colony formation was examined after culturing for 12 days. The average colony number was calculated and the colonies from 3 separate experiments were photographed. Bars represent means ± S.D. “*” Represent significant difference (*P* < 0.05) in *t*-test.

Previous studies have already shown that overexpression of YAP increases the anchorage-independent growth of MCF10A cells^49^ and knockdown of TAZ in MCF-7 cells reduces the colony formation and tumorigenicity of TAZ^50^. Therefore, in order to check the functional differences between the stable and dimerized YAP/TAZ to their non-dimerized forms, first we performed colony formation assay using H460 cells stably expressing WPI vector, YAP2L-WT, YAP2L-C343A, TAZ-WT or TAZ-3CA (Fig. 6A). Interestingly, we found that YAP2L-WT and TAZ-WT overexpressed cells showed significantly higher numbers of colonies as compared to their cysteine mutants (Fig. 6B and C). These results were further verified by anchorage-independent cell transformation assay using H460 stable cell lines as used in Fig. 6B. As shown in Fig. 6B, YAP2L-WT and TAZ-WT overexpressed cell lines showed significantly higher numbers of colonies in soft agar as compared to YAP2L-C343A and TAZ-3CA mutant (Fig. 6D and E). All together, these results indicate that the homodimerized YAP and TAZ are more stable and showed more oncogenic characteristics as compared to non-dimerized form.

## Discussion

YAP and TAZ are the major downstream regulators of the Hippo signalling pathway and are involved in different oncogenic^31,35,51–53^, and pro-apoptotic functions^54,55^. Although there are tremendous amount of studies which showed the different mechanisms (e.g. phosphorylation, dephosphorylation, and ubiquitination) for regulation of YAP/TAZ functions^27,41,56–58^, whether they are also regulated due to structural changes is largely unknown. Here, we provide the first evidence that YAP and its paralog TAZ can form homodimer and subsequently increases their stability and oncogenic activity. Similar to our findings, previous studies have shown that MST1, an upstream component of Hippo signalling pathway, also undergoes dimerization^44^. Further studies showed that dimerization of MST1 and MST2 are required for their kinase activity^45,59,60^. Most interestingly, recent studies showed that TAZ rather than YAP1 can form dimers that form heterotetramer with TEAD transcription factor, suggesting that TAZ dimer may interact with TEAD dimer to activate transcription^61,62^. Our findings give a detailed molecular mechanism of their observations (i.e. cysteine-mediated disulphide dimer formation; YAP2L rather than YAP1 or YAP2 form dimers) and their functional significance (i.e. increase protein stability and transformation). Our studies lead to significant implication of further investigating whether other components of Hippo pathway such as MER, Expanded, Kibra, LATS1/2, TEAD, and MOB also form dimers and regulate their activities and downstream signalling pathways. Most significantly, we found that YAP/TAZ both form homodimer through cysteine residue(s) (Figs 1–4), while MST forms dimers through its SARAH and kinase domains^45,60,63,64^, suggesting that different Hippo components may be dimerized and regulated in distinct ways. Therefore identification of novel mechanisms or motifs which help in YAP/TAZ dimerization will definitely provide a significant contribution in cancer biology.

YAP was first identified as a proline-rich phosphoprotein bound to the SH3 domain of Yes and Src protein tyrosine kinases^65^. There are eight isoforms of YAP protein in humans, which are distinguish from each other either by WW domain or activation domain^46,66^. Since many functional domains are overlapping in different YAP isoforms and most of the commercially available YAP antibodies cannot detect all of the isoforms, they can be only identified by qRT-PCR of their mRNAs. Therefore, the abundance and distinct functions of each YAP isoform are still largely unknown. Currently, three isoforms of YAP named YAP1, YAP2, and YAP2L have been used in most of the previous studies (Fig. 1A)^41,67^. YAP2 (also known as Yap1–2 gamma; Uniprot code: P46937–1) contains an extra WW domain than YAP1 (also named Yap1–1 gamma; Uniprot code: P46937–6). Although the exact functional difference between YAP1 and YAP2 has not been clearly elucidated, some differences have been reported. For example, YAP2 is considered as the canonical sequence of YAP is shown to be predominantly expressed in neural tissues^68^. In addition, YAP2 rather than YAP1 was shown to interact with p73^69^ and AMOTL1 (Angiomotin-Like-1)^55^. Moreover, YAP2 has been described to be a more potent transcriptional co-activator of ErbB-4 than YAP1^68^. However, the molecular mechanism underlying distinct function of YAP1 and YAP2 is unclear. Recent structural studies suggest that different selectivity of WW domains (WW1 vs WW2 in YAP2) may cause differential binding of YAP1 and YAP2 to their interacting proteins such as p73^70^.

Until now, the function of YAP2L remains elusive. YAP2L contains an extra AMRNINPSTANSPKCQ-amino acid motif than YAP2 (Fig. 1A). Interestingly, our results showed that the cysteine residue C343 in this extra motif is critical for dimerization of YAP2L both *in vitro* and *in vivo* (Fig. 3). Interestingly, we also noticed that YAP2L can form trimer and tetramers *in vitro* rather than *in vivo* in cells (Fig. 3). It has been suggested that although many proteins such as ASK1, PKA/G, and ATM can form dimers by forming disulphide bond through cysteine reside^71^, cells mostly contains a reducing condition which inhibits dimerization of molecules through disulphide bond formation^72^. It is possible that the condition for the formation of oligomers is more stringent than that of dimers. Therefore, they cannot form a high-order complex in more reducing condition in cells *in vivo*. Intriguingly, YAP paralog TAZ can only form dimers rather than oligomers through under non-reducing conditions *in vitro* with purified TAZ-GST (Fig. 4F) and *in vivo* with overexpressed TAZ (Fig. 4A–D) or endogenous TAZ (Fig. 4E). It is possible that all 3 cysteine residues are required for TAZ dimer formation, which limits its formation of high-order oligomer structure. Since cysteine residues are also important for the formation of heterodimers of a protein with other proteins, it will be very interesting to further explore how cysteine residues of YAP2L/TAZ interact with other proteins in signalling transduction. A proteomic screen using YAP-2L and YAP-C343A or TAZ and TAZ-3CA may identify novel cysteine-specific binding partners of YAP2L and TAZ and will provide a more comprehensive understanding the roles of YAP2L and TAZ in cancers in the future.

Many studies have already shown that post-translational modulation of YAP and TAZ play important roles in the regulation of their stability, activity and function^73^. It has been shown that YAP/TAZ can be regulated post-translationally by phosphorylation/dephosphorylation^41,42,74–80^, acetylation^81^, and ubiquitinastion/deubiquitination^27,42,82^, sumoylation^83^, and methylation^84^. Therefore, identification and characterization of post-translational modification of YAP/TAZ is very important for comprehensive understanding of their functions in cancer. Recent evidence suggests that cysteine residue can also undergo many types of post-translational modifications such as sulfhydration, S-Nitrosylation, S-Glutathionylation, and sulfenylation (SOH) and formation of disulfide bonds in the presence of reactive oxygen and nitrogen species (ROS and NOS), which results in different changes in the behaviour of cells^85^. Recently, it has been shown that the cysteine residues on TAZ can be S-Glutathionylated in the presence of oxidative stress ROS, which increases the stability and activates the transcriptional activity of TAZ^86^. Therefore, it is possible that oxidative stress may regulate TAZ or YAP2L stability and function by enhancing their dimerization through post-translational modification of cysteine residues. Since cysteine modification and redox system play important roles in cell signalling and various biological functions such as tumorigenesis and drug resistance^87–89^, it will very interesting to further explore how YAP2L/TAZ is modulated through cysteine in these biological processes.

We also realized that due to reducing condition in human cells, YAP2L and TAZ dimers only account for approximately 20–30% of total YAP2L/TAZ in cells (Figs 2A, 4B,E). This may be why moderate effects were observed when dimers were disrupted by cysteine(s) mutations in YAP2L/TAZ (Fig. 6). However, the proportion of YAP2L/TAZ will be significantly increased under redox stress condition due to post-translational modulation of cysteine residues in YAP2L/TAZ. It will be very interesting to further explore how dimerization of YAP2L/TAZ is regulated under various stress conditions.

Taken together, we have identified a novel mechanism of YAP2L/TAZ dimerization through disulphide bond formation of cysteine residues. We have shown that dimerization of YAP2L/TAZ play important roles in the regulation of their stability and oncogenic function. Further studies on the detail mechanisms and functional effect of this dimerization through post-translational modulation of cysteine residues in YAP2L/TAZ under various conditions will provide a significant contribution to our understanding of the roles of YAP2L/TAZ in cancer development and therapy.

## Experimental Procedures

### Plasmids construction and Transfection

Plasmids construction and site-directed mutagenesis were performed as described previously^41^. Full length cDNAs of human YAP1α (accession number NM_006106.4), YAP2α (accession number NM_001195044.1), YAP2γ/YAP2L (accession number NM_001130145.2), TAZ (accession number NM_001168278.2) and cysteine mutants of YAP2L or TAZ were subcloned into pcDNA3.1-hygro-3xFLAG, pcDNA3-HA, pGEX4T-1, HA-tagged WPI lentiviral vector or pET-23b (+) vectors, respectively. Transfections of plasmids into cells were carried out by using PolyJet^TM^ (SignaGen) according to the manufacturer’s protocol.

### Cell culture

Dulbecco’s modified eagle medium (DMEM), roswell park memorial institute-1640 (RPMI-1640) (1×), and fetal bovine serum (FBS) were purchased from Invitrogen (Carlsbad, CA). HEK293 (human embryonic kidney cells) were cultured in Dulbecco’s Modified Eagle’s Medium (DMEM; Sigma, #D6429) supplemented with 10% fetal bovine serum and 1% penicillin/streptomycin (P/S) (Invitrogen). H460 (human NSCLC cells) were maintained in RPMI- 1640 medium (Sigma, #8758) containing 10% FBS and 1% P/S supplemented with Sodium Pyruvate (1 mM), HEPES (10 mM) and Glucose (2.5 mg/ml). Cells were cultured at 37 °C in humidified air containing 5% CO_2._

### Lentivirus production, infection and establishment of stable cell lines

Lentivirus production and concentration were carried out as described before^90^. For infection of H460 cells with lentivirus, 2 × 10^5^ cells were plated into each well of a 6-well plate. After 24 hrs of plating cells were infected with WPI, WPI-HA-YAP-WT, and WPI-HA-YAP-C343A, WPI-HA-TAZ-WT or WPI-HA-TAZ-3CA (C262A, C320A, C363A) lentivirus respectively using 8 µg/ml of Polybrene in each well. 24 hrs after infection, cells were selected with 200 µg/mL Hygromycin B. The expression of respective genes was confirmed by western blot using anti-HA antibody.

### Fusion protein production and GST-pull-down assays

GST fusion proteins were produced and purified as described previously^91^. For Purification of His-Tag protein, YAP2L-WT and YAP2L-C343A cloned in pET-23b (+) vectors were transformed into BL21 (RIPL) competent cells. The proteins were induced by incubating the medium containing bacteria with 1 mM isopropyl β-D-thiogalactoside (IPTG) for overnight at 20 °C, and were subsequently purified by passing the bacterial lysate through HisTrap HP histidine-tagged protein purification columns. For GST-pull down assays, 200 µg of respective protein lysates were pre-cleared overnight at 4 °C with 20 µL glutathione sepharose 4B beads. Next day, 5 µg of appropriate GST or GST fusion proteins were added to the corresponding supernatants and incubated at 4 °C for 2 hr. 20 µL of 50% GSB was then added and further incubated for 1hr. After this, beads were washed four times with lysis buffer (50 mM Tris-HCl pH 7.4, 150 mM NaCl, 1 mM EDTA and 1.0% Nonidet P-40) and 2xSDS sample buffer was added to beads and boiled for 10 mins and centrifuged. The supernatants were run on SDS-PAGE and blotted with respective antibodies.

### Antibodies and reagents

Antibodies against YAP and HA-F7 were purchased from Santa Cruz Biotechnology (Santa Cruz, CA). Anti–FLAG-M2 and β-actin antibodies were from Sigma Aldrich. TAZ antibody was from BD Transduction Laboratories. Anti-His-HRP antibody was obtained from Miltenyi Biotec Inc. Restriction enzymes for cloning were purchased from NEB (New England Bio lab). HisTrap^™^ and GSTrap^™^ Fast Flow columns were from GE Healthcare. Protein G/A-Agarose was purchased from Roche Diagonistics.

### Anchorage-independent cell transformation assay

Soft agar assay was performed as described before^41^. Briefly, triplicates of different H460 stable cells (2 × 10^3^) were mixed with complete growth media containing 0.4% agarose and then plated over 0.8% agarose in each well of 6-well plates. After 24 hr, 1 ml of complete growth medium with 50 μg/ml of Hygromycin B was added to each well. Culture media was refreshed at every 3 days. The cultures were maintained at 37 °C in a 5% CO_2_ incubator for 15 days and colonies were stained with 0.005% crystal violet in 20% methanol. Pictures were taken by using Bio-Rad Gel Doc System (Bio-Rad, Mississauga, Canada) and colonies were quantified by colony count program in Quantity One software. Data from soft agar assays were statistically analyzed using unpaired t-tests, and *P* values < 0.05 were considered significant.

### Colony formation assay

Triplicate of different H460 stable cell lines were plated in 6-well plates (500 cells/plate) in triplicate. The cells were incubated at 37 °C in humidified air containing 5% CO_2_. The media was refreshed at every 3–4 days. After 12 days, the colonies were fixed with 95% methanol and stained with 0.5% crystal violet in 20% methanol. Colonies were counted and quantified as described above.

### Immunoblotting and Co-immunoprecipitation (CO-IP)

Immunoblotting and CO-IP was carried out as described previously^41^. In brief cells grown to 70% to 80% confluency were harvested in RIPA/1%-NP40 lysis buffer. Protein samples were subjected to SDS-PAGE and immunoblotted with respective antibodies using standard protocol. To check dimerization of YAP/TAZ, 15 μg of cell lysate or 1 μg of purified His-YAP-WT/-YAP-C343A or GST-TAZ-WT/-TAZ-3CA were mixed with 2 μl of 5xSDS sample buffer with/without DTT and boiled for 3 min at 60 °C and subjected to SDS-PAGE and immunoblotted with respective antibodies. For co-immunoprecipitation, cells expressing different genes were harvested in 1% NP-40 lysis buffer. After checking the expression of different proteins, equal amounts of protein were precleared overnight at 4 °C using Protein-A/G-agarose. Then the supernatant were subjected to immunoprecipitation using anti-HA-F7/FLAG-M2 antibody for 2 hr at 4 °C. After 2hr, 20 μl of protein- A/G-agarose was added for another 1 hr and beads were washed with 150mM-Nacl-1% NP-40 lysis buffer. Then 20 μl of 2xSDS sample buffer was added to beads and boiled for 10 mins and centrifuged. The supernatants were run in SDS-PAGE and blotted with respective antibodies.

## Electronic supplementary material

Supplementary Information
