# Supplementary material for: Cysteine residues are essential for dimerization of Hippo pathway components YAP2L and TAZ
Source: Sci Rep. 2018 Feb 22;8:3485. doi: 10.1038/s41598-018-21828-6 (PMC5823869; doi:10.1038/s41598-018-21828-6)

SUPPLEMENTARY INFORMATION

Cysteine residues are essential for dimerization of Hippo pathway components YAP2L and TAZ

Prem Khanal[1], Zongchao Jia[2] and Xiaolong Yang[1]

[1]Department of Pathology and Molecular Medicine, Queen's University, Canada
[2]Department of Biomedical and Molecular Sciences, Queen's University, Canada

To whom correspondence should be addressed: Xiaolong Yang, Ph.D., Richardson Lab 201D, 88 Stuart Street, Kingston, ON K7L 3N6, Canada; Tel: 613 533-6000 Ext.75998, Fax: Fax: 1-613-533-2970. E-mail: yangx@queensu.ca

Keywords: Cysteine, Hippo pathway, YAP, TAZ, dimerization, and protein stability

Supplementary Figure 1. (A) Original blots used in Fig. 1B. Images were taken using Amersham Imager from GE-Health care. (B) Original Blots used in Fig. 1C. Images were taken using Amersham Imager from GE-Health care.

(**A**)

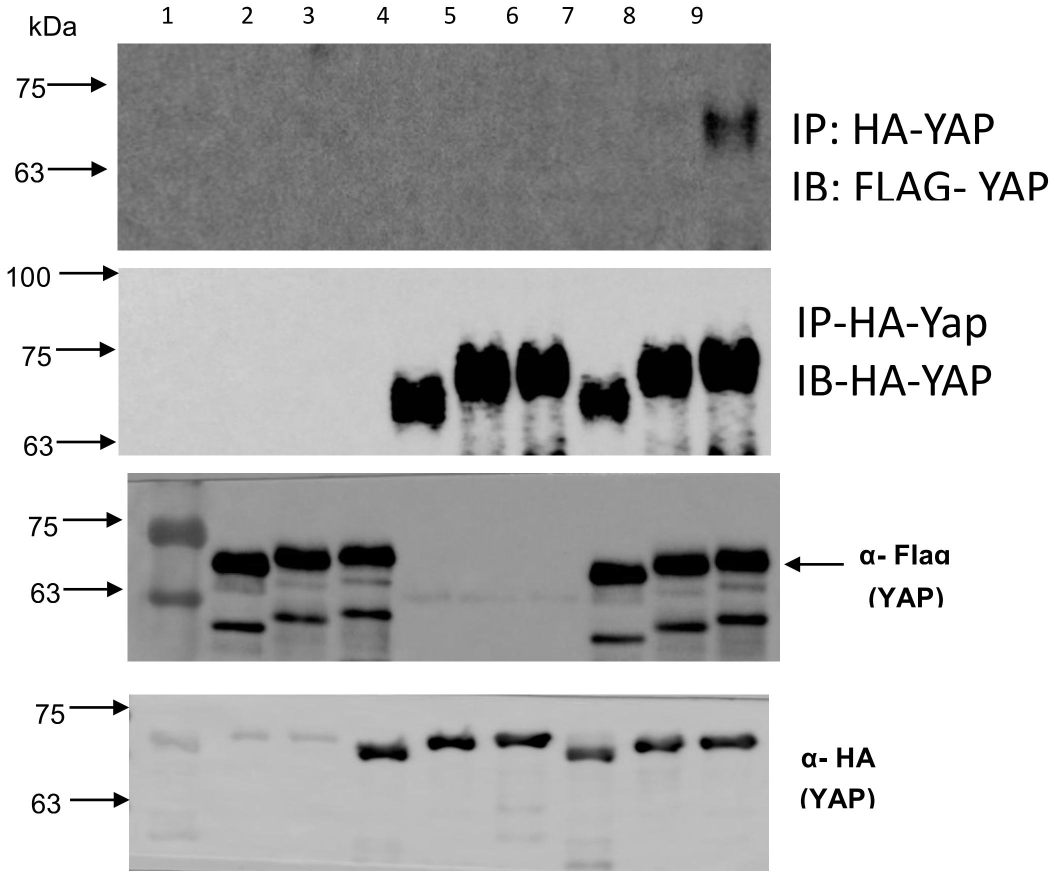

(**B**)

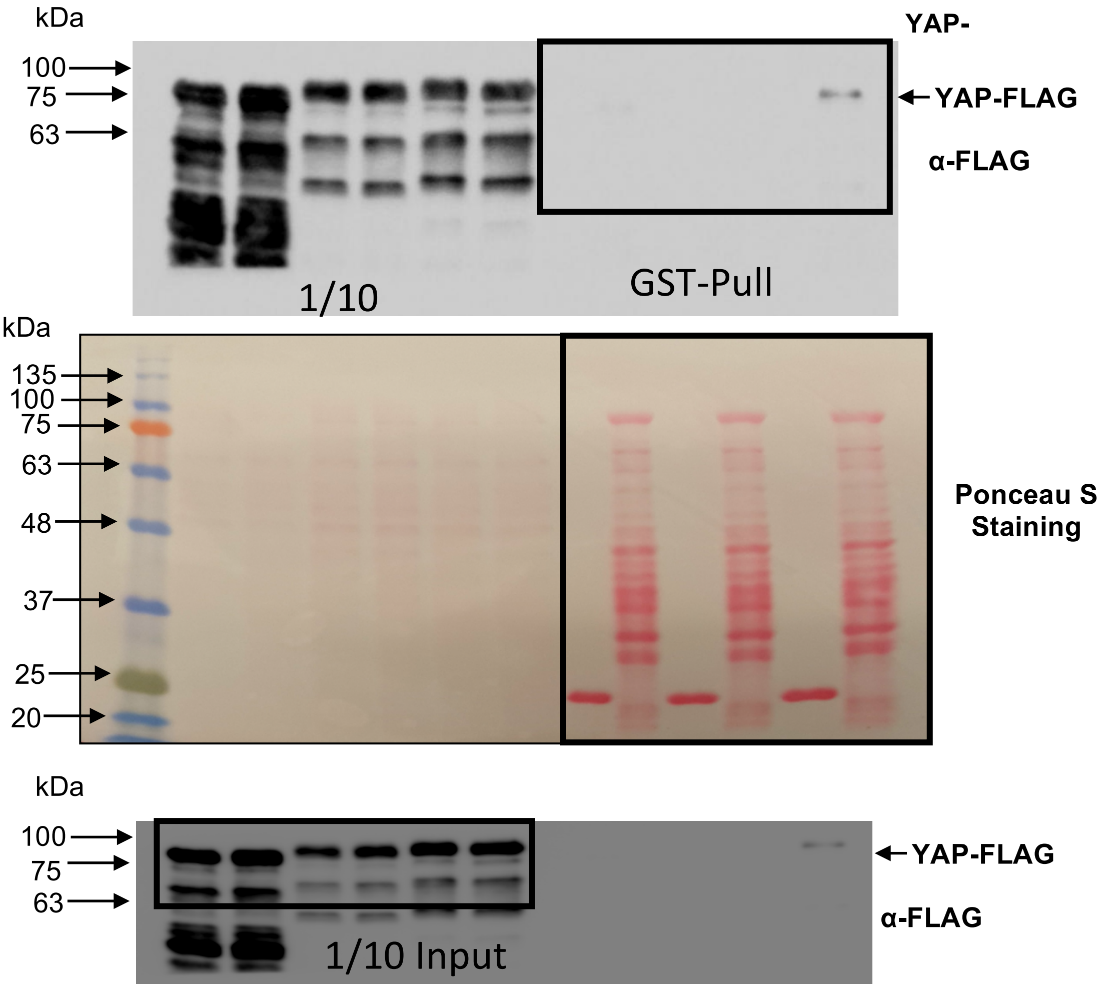

Supplementary Figure 2. (A) Original blots used in Fig. 2A. (B) Original blots used in Fig. 2B. Images were taken using Amersham Imager from GE-Health care. (C) Full blots of crop blots used in Fig.2C, The parsts of blots used in figure are highlighted with in square. (D) Full blots of crop blots used in Fig.2D. (E) original blots used in Fig. 2E.

(**A**)

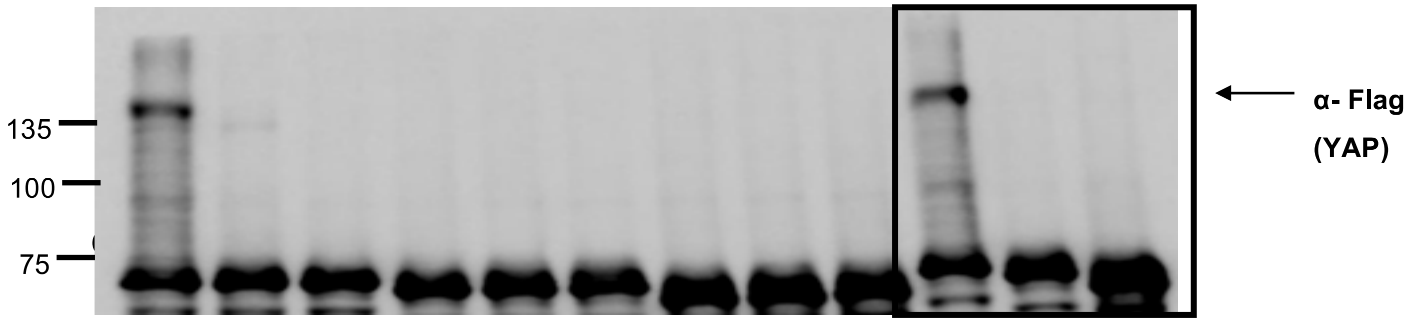

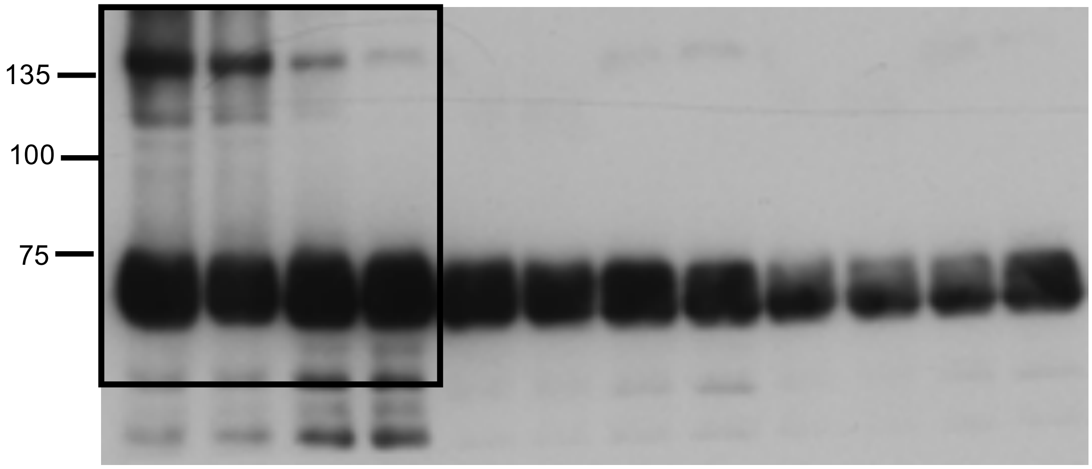
135
100
75

(**C**)

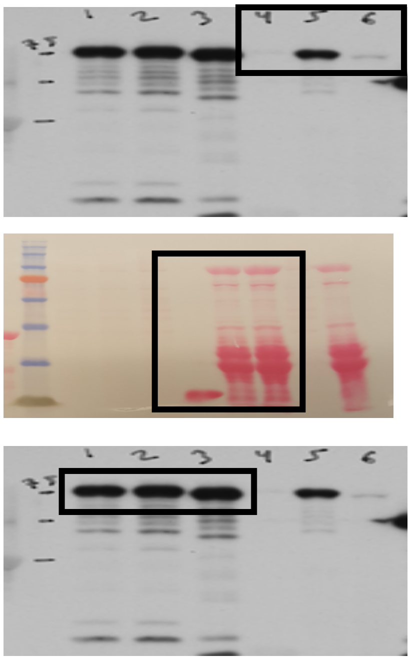
1 2 3 4 5 6
75

(**D**)

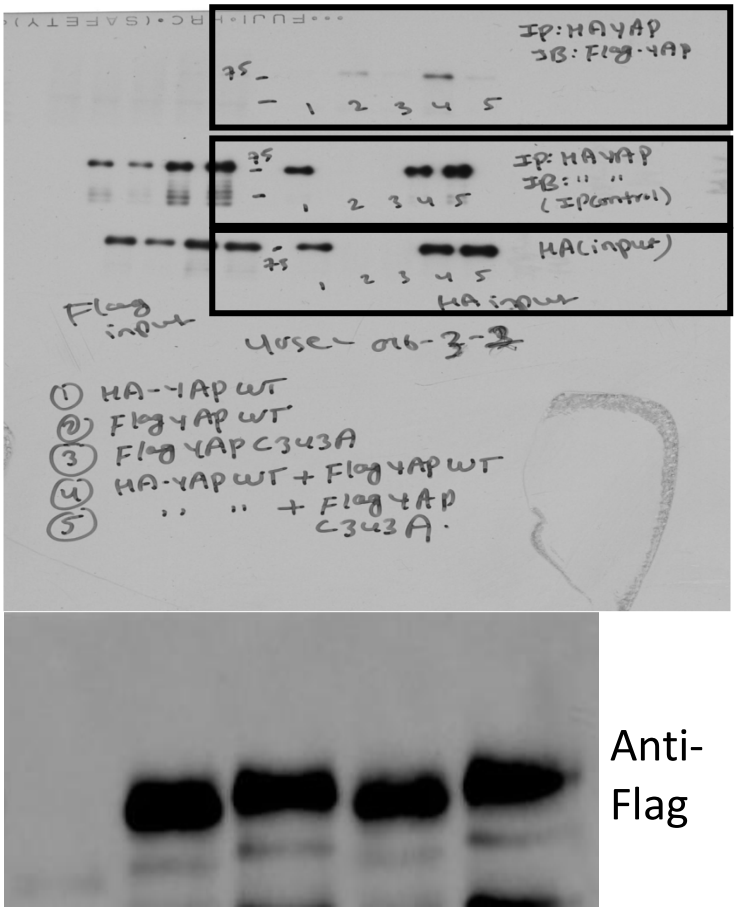
IP: HA YAP
IB: Flag-YAP
75
1 2 3 4 5
IP: HA YAP
IB: " "
(IP control)
HA (input)
HA input
Flag input
① HA-YAP WT
② Flag YAP WT
③ Flag YAP C343A
④ HA-YAP WT + Flag YAP WT
⑤ " " + Flag YAP C343A.

Anti-Flag

(E)

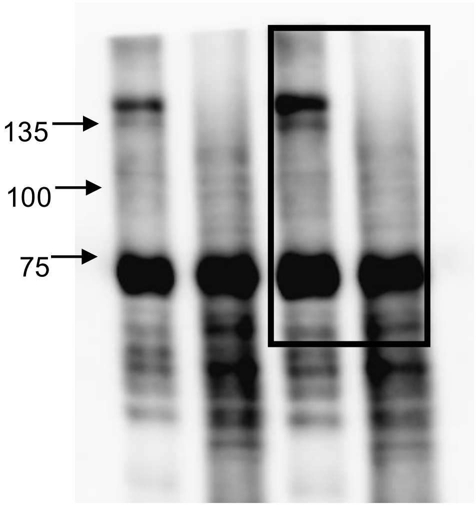

Supplementary Figure 3. (A) Original blots used in Fig. 3A. (B) Original blots used in Fig. 3B. (C) original blots used in Fig.3C. (D) Original blots used in Fig.3D.

(A)

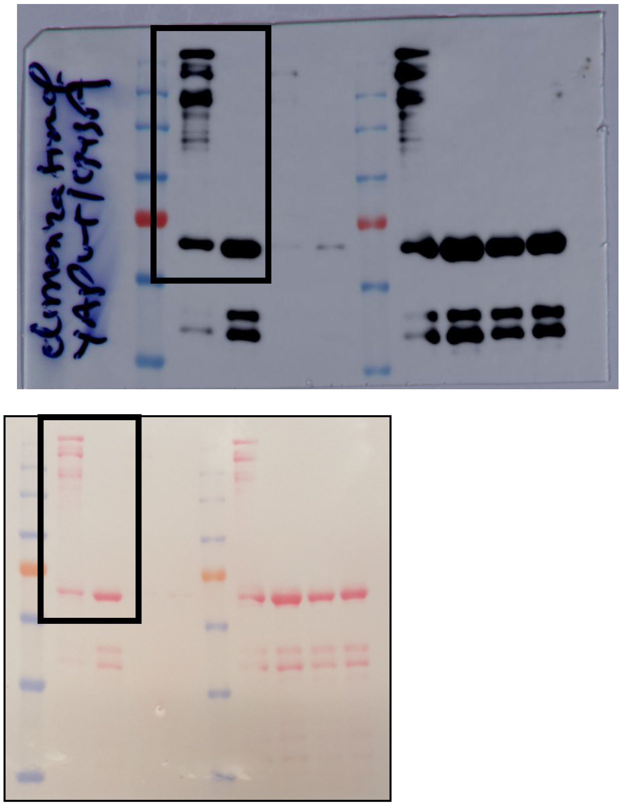

(B)

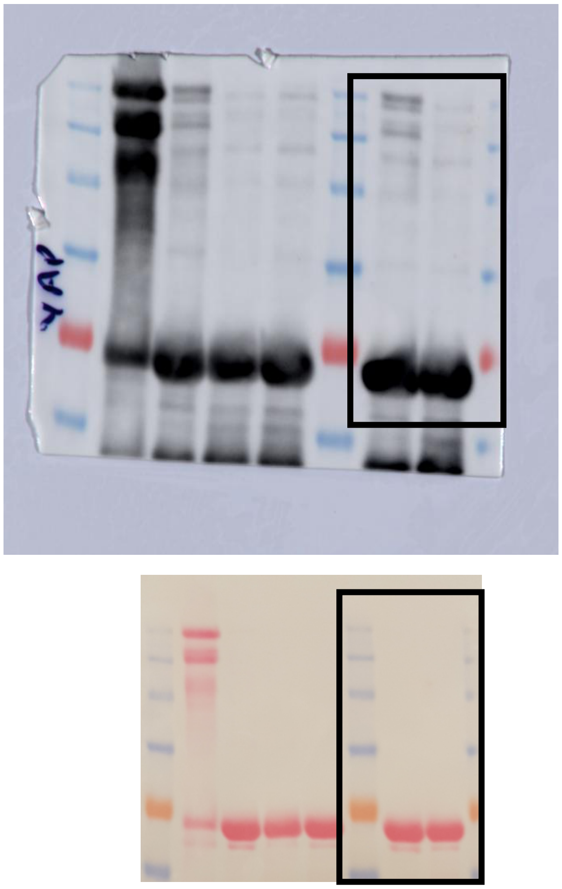

(C)

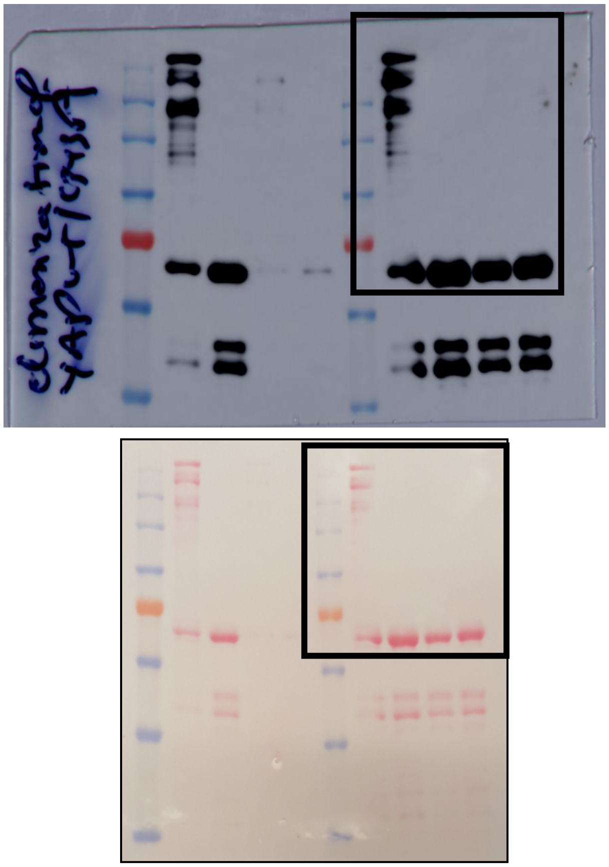

(D)

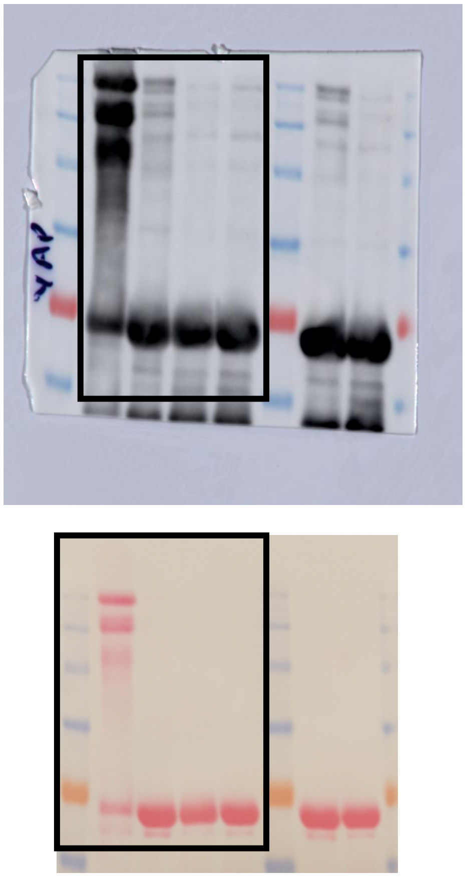

Supplementary Figure 4. (A) Original blots used in Fig. 4A. (B) Original blots used in Fig. 4B. (C) Original blots used in Fig. 4C. (D) Original blots used in Fig. 4D. (E) Original blots used in Fig. 4E

(A)

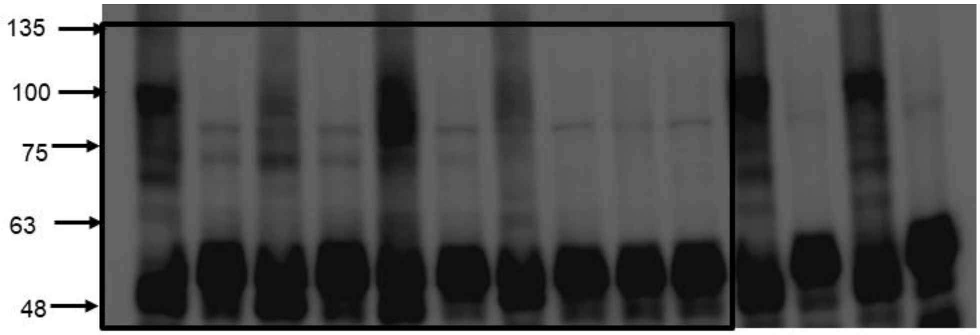

(B)

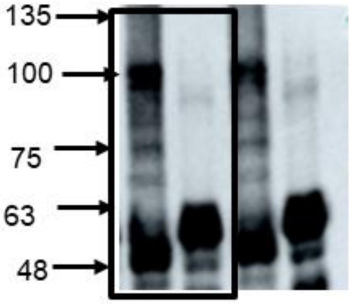

(C)

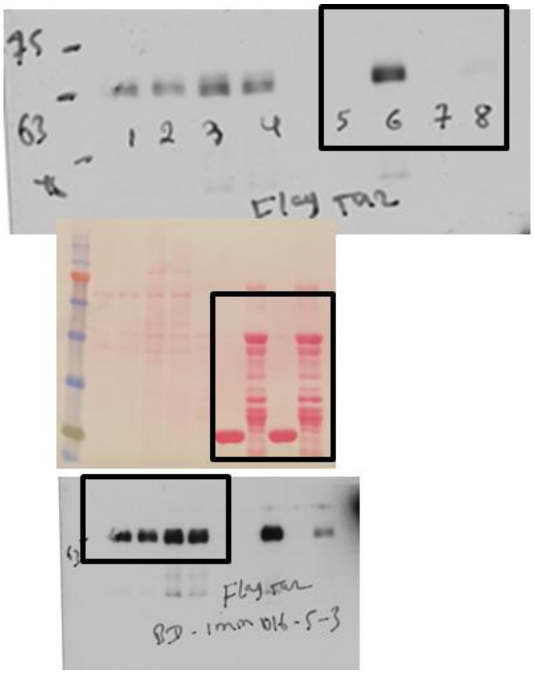

(D)

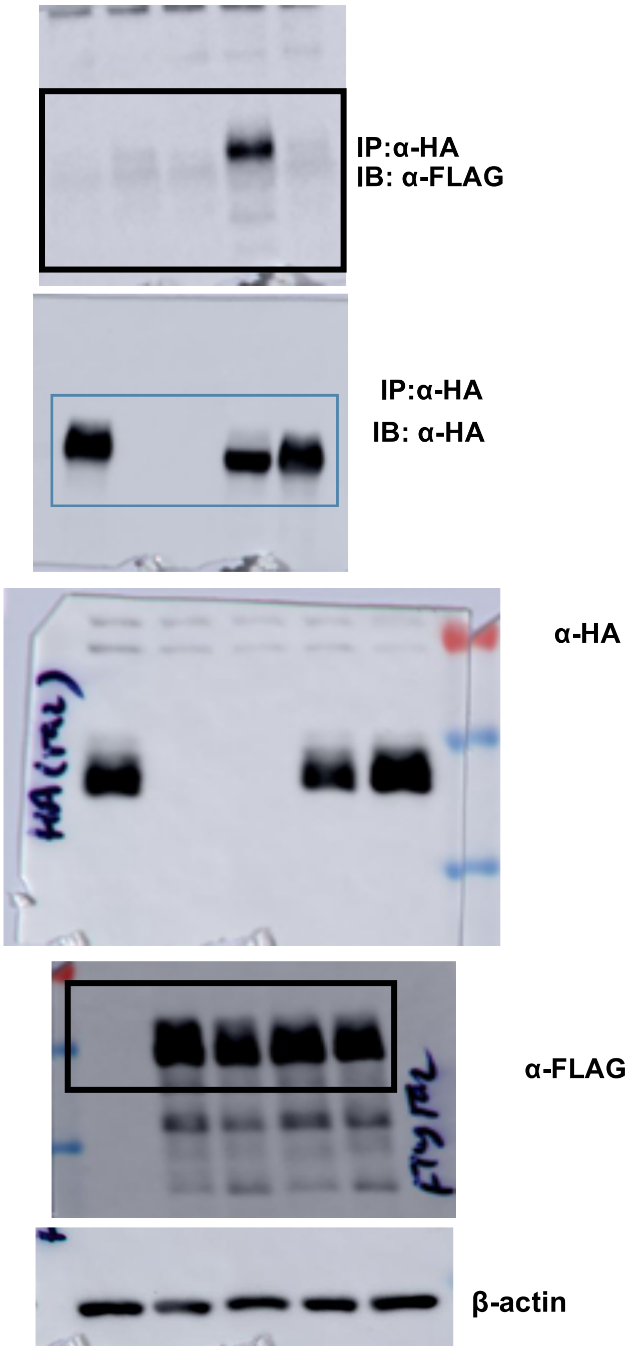

(E)

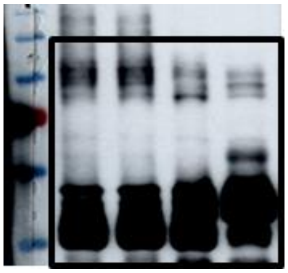

Supplementary Figure 5. (A) Original blots used in Fig. 5A. (B) Original blots used in Fig. 5B. (C) Original blots used in Fig. 5C. (D) Original blots used in Fig. 5D.

(A)

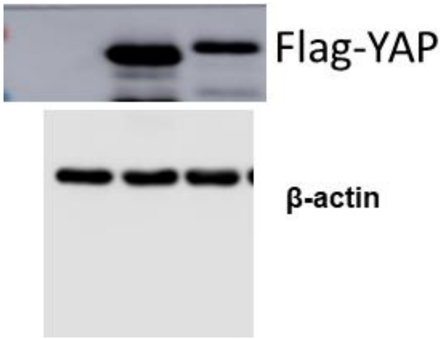

(B)

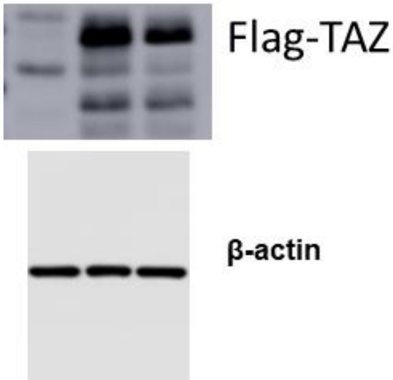

(C)

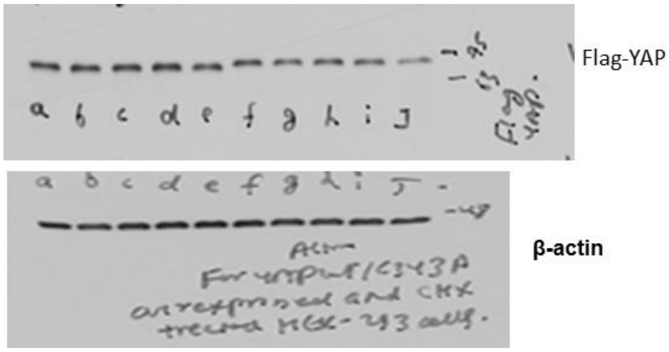

(D)

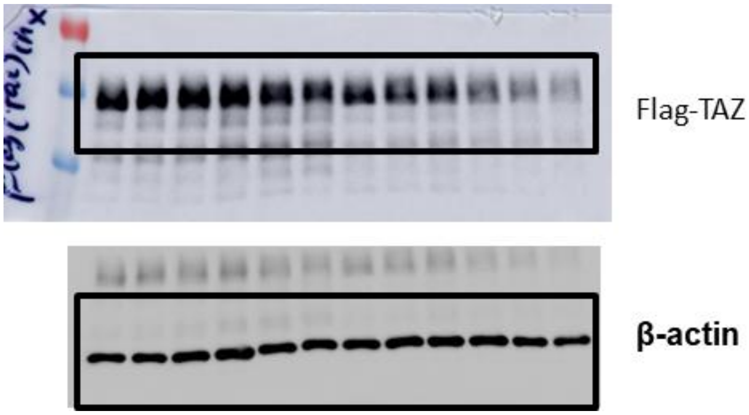

Supplementary Figure 6. (A) Original blots used in Fig. 6A.

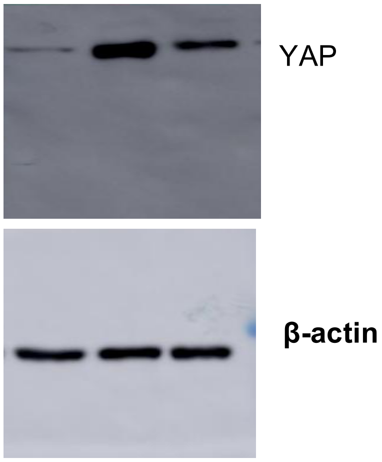

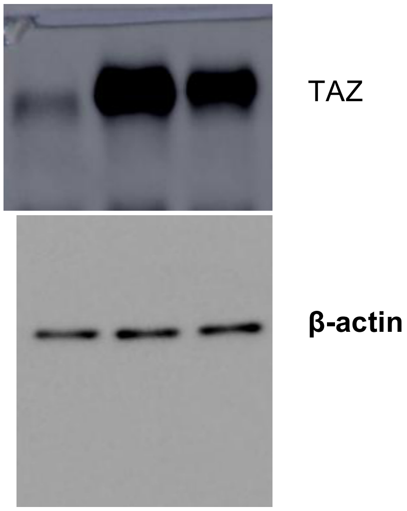

Supplement: Supplementary file 1 — Supplementary Information [file 41598_2018_21828_MOESM1_ESM.pdf]
